# Genetic and clinical phenotype of Dent disease in Chinese children and the etiological analysis of early - onset chronic kidney disease

**DOI:** 10.1186/s13052-025-02166-6

**Published:** 2025-12-08

**Authors:** Lanqi Zhou, Zuowei Yu, Yuan Yang, Yanxinli Han, Liru Qiu, Yu Zhang, Fengjie Yang, Jianhua Zhou

**Affiliations:** Department of Pediatrics, Tongji Hospital, Tongji Medical College, Huazhong University of Science and Technology, Wuhan, Hubei province 430030 China

**Keywords:** Dent disease, *CLCN5*, *OCRL*, Chronic kidney disease

## Abstract

**Background:**

A prominent feature of Dent disease (DD) is the progressive decline in renal function, with 30% - 80% of male patients advancing to end-stage renal disease between the ages of 30 and 50 years. However, limited research exists on the chronic kidney disease (CKD) progression in pediatric patients with DD. This study aimed to retrospectively analyze the clinical features, genetic variant spectrum, and prognosis of pediatric patients with DD and explore the factors associated with early renal failure during childhood in these patients.

**Methods:**

We analyzed the genetic backgrounds, clinical phenotypes, and laboratory data of 23 unrelated patients with DD.

**Results:**

All patients were males with low-molecular-weight proteinuria. *CLCN5* variants were detected in 19 patients (Dent disease type 1, DD1), and *OCRL* variants were identified in 4 patients (Dent disease type 2, DD2). Sixteen mutations have not been reported previously. During follow-up, progression to CKD was documented in 7 patients: 6 with DD1 and 1 with DD2. CKD stages were distributed as follows: 4 patients at stage II, 2 at stage III, and 1 at stage V. Nephrolithiasis (100% vs 30.76%, *P =* 0.011), nephrocalcinosis (85.33% vs 15.38%, *P* = 0.010), and acute kideny injury (100% vs 0%, *P <* 0.001) were significantly more common in those CKD patients with DD1.

**Conclusion:**

This study expands the genetic spectrum of Dent disease and highlights that some pediatric patients may progress to CKD during childhood. CKD progression may be associated with early occurrences of nephrolithiasis, nephrocalcinosis, and acute kidney injury.

## Introduction

Dent disease (DD) is a rare X-linked recessive monogenic disorder characterized by proximal renal tubular dysfunction. Key characteristics include low-molecular-weight proteinuria (LMWP), hypercalciuria, nephrolithiasis, nephrocalcinosis and progressive renal failure [1, 2]. First identified in 1964, DD is classified as two primary subtypes based on the causative gene, Dent disease type I (OMIM #300009) or Dent disease type II (OMIM #300555). Approximately 60% of cases are attributed to pathogenic variants in the *CLCN5* gene, which characterize Dent disease type 1 (DD1), while 15% are associated with pathogenic variants in the *OCRL* gene, defining Dent disease type 2 (DD2) [3, 4]. Notably, pathogenic variants remain unidentified in 25% of patients [5, 6].

The *CLCN5* gene, located on chromosome Xp11.22, encodes the electrogenic chloride/proton exchanger ClC-5. It is predominantly expressed in proximal tubular cells of the kidney and is crucial for renal endocytosis [7, 8]. ClC-5 facilitates endosomal acidification by exchanging chloride ions (Cl¯) for protons (H^+^), which supports the H^+^-ATPase activity. This exchange maintains Cl¯ homeostasis within endosomes, essential for effective endocytosis. Variants in the *CLCN5* gene can significantly impair renal function, underscoring the importance of this Cl¯/H^+^ exchanger in kidney physiology [9]. Similarly, the *OCRL* gene, located on chromosome Xq26.1, encodes a phosphatidylinositol bisphosphate 5-phosphatase [10, 11].This protein, expressed in the trans-Golgi network, glomeruli, and all tubular segments, regulates membrane transport, cytoskeletal dynamics, and vesicular trafficking [4].

A prominent feature of DD is the progressive decline in renal function, with 30% to 80% of male patients advancing to end-stage renal disease (ESRD) between the ages of 30 and 50 years [12–14]. However, limited research exists on tchronic kidney disease (CKD) progression in pediatric patients with DD. To date, European and Chinese cohorts have reported 44 and 4 cases, respectively, of childhood CKD associated with DD [15, 16], although these cases lack detailed descriptions. In this study, we retrospectively analyzed the clinical characteristics, genetic variant spectrum, and follow-up data of 23 children with DD. The objective was to investigate the factors influencing CKD progression in these patients during childhood and to increase pediatrician awareness’ and understanding of this rare disease.

## Materials and methods

### Patients

We retrospectively analyzed 23 patients diagnosed with DD between 2012 and 2024 in the Department of Pediatrics, Tongji Hospital, Tongji Medical College, Huazhong University of Science and Technology. The inclusion criteria for patients were as follows: (1) LMWP, and (2) pathogenic variant detection in *CLCN5* or *OCRL* by next-generation sequencing or Sanger sequencing. Patients meeting criteria (1) and (2) [17], were diagnosed with DD. The exclusion criteria were as follows: onset of disease after the age of 18 years, Lowe syndrome diagnosis, or incomplete medical records [17].

### Genetic testing

Whole-exome sequencing and bioinformatics analysis were performed at Chigene Translational Medicine Research Center. After informed consent was obtained, blood samples were collected from patients and their relatives. Genomic DNA was isolated from the samples, and quality assessment was performed. Target fragments were enriched, and an exome library was constructed using the xGen Exome Research Panel v2.0 (IDT, Iowa, USA) with capture probes and liquid hybridization. High-throughput sequencing was performed using an MGISEQ-T7 series sequencer, achieving at least 99% coverage of the target sequences. Raw sequencing data were processed using Fastp to remove adapters and filter out low-quality reads. Paired-end reads were aligned to the Ensemble GRCh37/hg19 reference genome using Burrows-Wheeler Aligner. Base quality score recalibration and the calling for single nucleotide variants and short insertions or deletions (indels) were performed using GATK. Variants with high confidence were identified based on sequence depth and variant quality. qPCR validation of large deletions was performed. Variants were evaluated for pathogenicity based on the standards set by the American College of Medical Genetics and Genomics [18].

### Clinical data

We collected clinical information on the patient, including demographics, clinical presentation, laboratory results, histopathological findings, follow-up examinations, and outcomes. LMWP was defined as protein electrophoresis showing low-molecular-weight protein accounts for more than 50%, or the low-molecular-weight protein in urine is at least 5 times higher. Hypercalciuria was defined as the increased urinary calcium excretion > 0.1 mmol/kg/day [16]. CKD was defined and staged according to the Kidney Disease Improving Global Outcomes (KDIGO) criteria [19]. Acute kidney injury (AKI) was defined based on serum creatinine measurements following the KDIGO creatinine criteria [20].

### Statistical analysis

Categorical data are presented as frequencies (%) and analyzed using chi-square or Fisher’s exact tests. Continuous variables with normal distribution are summarized as mean ± standard deviation (SD), whereas non-normally distributed data are reported as median (interquartile range [IQR]). Independent t-tests or Mann-Whitney U tests, as appropriate, were employed for group comparisons.

## Results

### Clinical phenotypes

All pediatric patients in this cohort were males. Of the 23 patients, 19 (82.6%) were diagnosed with DD1, and 4 (17.3%) with DD2. The phenotypic characteristics at clinical diagnosis for patients with DD1 and DD2 are illustrated in Table 1. LMWP incidence was 100.0% in all patients. Although nephrotic-range proteinuria prevalence appeared numerically higher in DD2 (50.0% vs. 5.26% in DD1), this observation requires validation in larger cohorts given the small DD2 sample size (Table 2). Kidney failure was observed in one patient with DD1 at the age of 5 years and one patient with DD2 at the age of 15 years at diagnosis. Renal biopsies were performed on 9 patients with DD1 and 1 patient with DD2, with results detailed in Table 1.

**Table 1:** Clinical and genetic characteristics of patients with DD at diagnosis

Patients ID	Sex	Age at diagnosis (years)	Family history	Serum K (mmol/L)	Serum P (mmol/L)	Serum creatinine (umol/L)	Urine albumin/ creatinine ratio (ug/mg)	24-hour urinary Calcium (mmol/kg)	Proteinuria	Hematuria	Glycosuria	Nephrolithiasis	Nephrocalcinosis	Kidney failure	Gene	Nucleotide	Protein	Type of mutation	ACMG classification	renal pathology
1	Male	9	No	3.83	1.27	39	327.3	0.08	Yes	Yes	No	No	No	No	CLCN5	c.1909C > T	p.Arg637*	Nonsense	PVS1 + PS4 + PM2	FSGS
2	Male	3	Yes	4.33	1.44	19	285.4	0.05	Yes	No	No	No	No	No	CLCN5	c.656 G > A	p.Cys219Tyr	Missense	PM1 + PM5 + PM2 + PP3	-
3	Male	3	No	4.03	1.55	30	236.5	0.14	Yes	Yes	No	No	No	No	CLCN5	c.1517_1518delTA	p.Ile506fsTer22	Frameshift	PVS1 + PM2	-
4	Male	1	No	4.74	1.72	23	591.7	0.22	Yes	Yes	No	Yes	Yes	No	CLCN5	c.1272delG	p.Lys424Asnfs*5	Frameshift	PVS1 + PM2	-
5	Male	8	No	4.41	1.45	27	204.5	0.05	Yes	No	No	No	No	No	CLCN5	c.299A > G	p.His100Arg	Missense	PM2 + PP2	-
6	Male	5	No	3.75	1.15	38	378.2	0.15	Yes	Yes	No	No	No	No	CLCN5	c.2110C > T	p.Arg704*	Nonsense	PSV1 + PM2 + PP5	MCD
7	Male	2	No	4.71	1.67	30	322.2	0.095	Yes	Yes	Yes	No	No	No	CLCN5	c.2362C > T	p.Arg788*	Nonsense	PSV1 + PM2 + PP5	-
8	Male	4	No	2.64	0.55	30	954.1	0.17	Yes	Yes	No	No	No	No	CLCN5	c.941C > T	p.Ser314Leu	Missense	PP3 + PP5 + PP2 + PM5	MsPG
9	Male	12	No	4.20	1.69	22	732	0.165	Yes	Yes	No	No	No	No	CLCN5	c.674dupT	p.Ser226Ilefs*17	Frameshift	PVS1 + PM2	MCD
10	Male	8	Yes	4.24	1.17	34	243.3	0.290	Yes	No	No	No	No	No	CLCN5	c.1039A > T	p.Thr347Ser	Missense	PM1 + PM2 + PP3	MsPG
11	Male	5	No	3.80	1.25	31	613.5	-	Yes	Yes	No	Yes	No	No	CLCN5	c.478 T > C	p.Cys160Arg	Missense	PP2 + PP3 + PM2	-
12	Male	14	Yes	2.94	0.62	43	373.1	0.120	Yes	No	No	Yes	No	No	CLCN5	c.1164_1167dup	p.Asn390Valfs*2	Frameshift	PVS1 + PM2	FSGS
13	Male	6	No	4.25	1.41	96	404.8	0.13	Yes	Yes	No	Yes	No	Yes	CLCN5	c.1566_1568del	p.Val523del	Deletion	PM1 + PP5 + PM4 + PM2	FSGS
14	Male	10	Yes	4.08	1.24	45	269.1	0.17	Yes	Yes	No	No	No	No	CLCN5	c.1039C > T	p.Arg347*	Nonsense	PVS1 + PP5 + PM2	-
15	Male	12	No	3.24	1.06	66	563.3	0.395	Yes	Yes	No	Yes	No	No	CLCN5	c.992_993insAGTATTAT	p.Phe334Xfs*1	Frameshift	PVS1 + PM2	-
16	Male	2	No	4.41	1.52	22	230.9	0.04	Yes	Yes	No	No	No	No	CLCN5	c.293 G > C	p.Arg98Pro	Missense	PM2 + PP2 + PP3	-
17	Male	3	No	4.18	1.32	32	223.9	0.04	Yes	No	Yes	No	No	No	CLCN5	del(EXON:5–10)		Deletion	PVS1 + PM2	MCD
18	Male	4	Yes	3.90	1.31	26	328.8	0.16	Yes	Yes	No	No	No	No	CLCN5	C.726 + 5 G > C		Splicing	PM2 + PP3	-
19	Male	3	No	4.46	1.67	28	471.12	0.20	Yes	No	No	No	No	No	CLCN5	C.1205 G > A	p.Trp402*	Nonsense	PVS1 + PS2 + PM2	-
20	Male	6	No	3.46	0.62	28	698.1	0.188	Yes	No	No	No	No	No	OCRL	c.1040 G > A	p.Gly347Glu	Missense	PP3 + PM1 + PP5 + PM2	-
21	Male	6	No	4.09	1.08	27	-	0.02	Yes	Yes	No	No	No	No	OCRL	c.808 G > A	p.Asp270Asn	Missense	PP3 + PM2 + PM1	-
22	Male	15	No	3.41	1.46	112	236.9	0.036	Yes	Yes	No	No	No	Yes	OCRL	c.2431 T > C	p.Cys811Arg	Missense	PM1 + PM2 + PP3	MCD
23	Male	3	Yes	4.62	1.76	28	915.4	0.40	Yes	Yes	No	Yes	No	No	OCRL	C.461delA	p.Asn154Ilefs*5	Frameshift	PVS1 + PM2	-

DD: dent disease; FSGS: focal segmental glomerulosclerosis; MCD: minimal change disease; MsPG: mesangial proliferative glomerulonephritis

**Table 2:** Clinical manifestation in patients with DD at diagnosis

	DD1 (n = 19)	DD2 (n = 4)
Age at diagnosis, years (mean ± SD)	5.99 ± 3.854	7.44 ± 5.222
Rickets, n (%)	3/19 (15.79%)	2/4 (50.00%)
Growth deficiency, n (%)	4/13 (30.77%)	3/3 (100%)
Nephrolithiasis, n (%)	5/19 (26.32%)	1/4 (25.00%)
Nephrocalcinosis, n (%)	1/19 (5.26%)	0/4 (0.0%)
Low molecular weight proteinuria, n (%)	19/19 (100%)	4/4 (100%)
hypercalciuria, n (%)	12/19 (63.16%)	2/4 (50.00%)
Glycosuria, n (%)	2/19 (10.53%)	0/4 (0.00%)
Hematuria, n (%)	13/19 (68.42%)	3/4 (75.00%)
Nephrotic range proteinuria, n (%)	1/19 (5.26%)	2/4 (50.00%)
Hypokalemia, n (%)	3/19 (15.79%)	2/4 (50.00%)
Hypophosphatemia, n (%)	2/19 (10.53%)	1/4 (25.00%)
Kidney failure, n (%)	1/19 (5.26%)	1/4 (25.00%)

DD: dent disease; DD1: dent disease type 1; DD2: dent disease type 2

### Genetic features

**Fig. 1:**
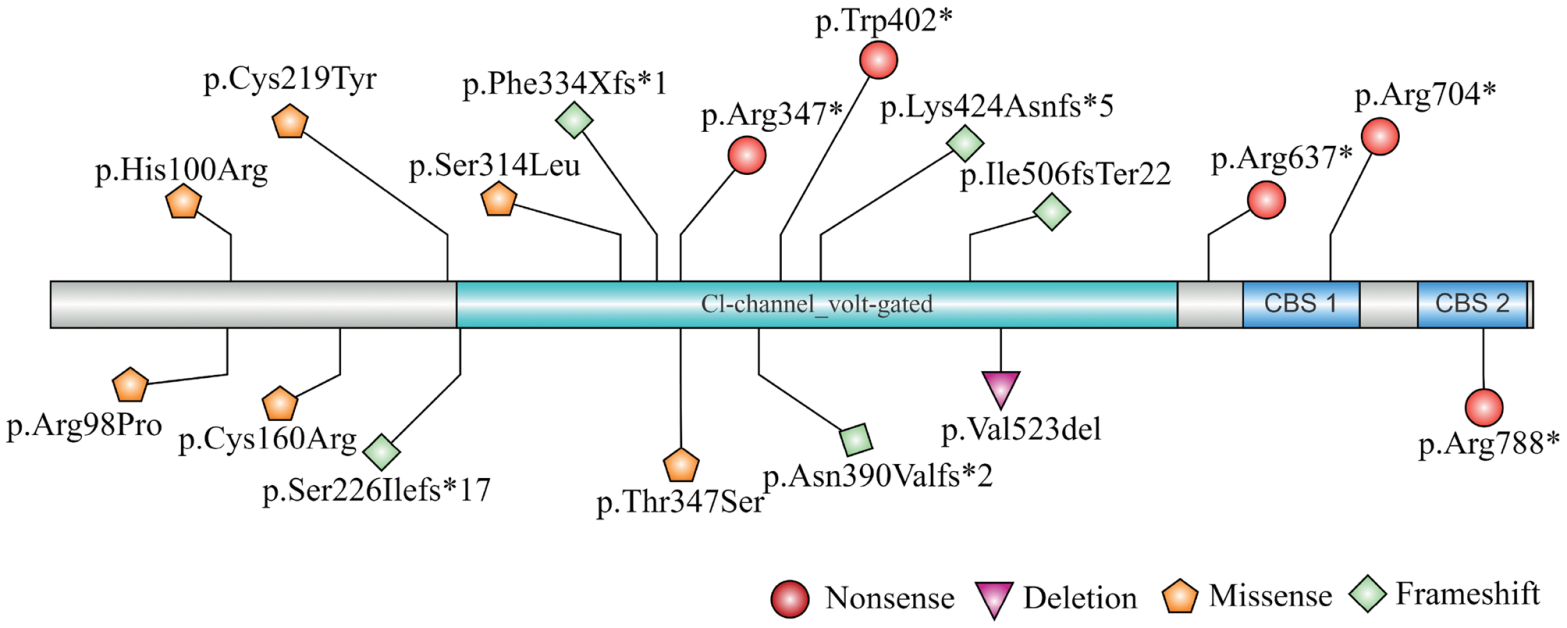
A schematic depicting the variants in CLCN5. The two variants, CLCN5 exon5-exon10del and CLCN5 c.726 + 5 G > C, are not shown in the figure

All 23 patients underwent genetic testing. *CLCN5* variants were detected in 19 patients, and *OCRL* variants were identified in 4 patients. In total, distinct variants were identified in 23 families (Table 1). For *CLCN5*, most variants were missense (31.6%), followed by frameshift (26.3%), nonsense (26.3%), deletion (10.5%), and splicing mutation (5.3%). Of these, 13 variants were novel. The identified variants were distributed across the CLCN5 gene (Fig. 1). The IBS Online Drawing Tool was used to create Fig. 1 [21]. For *OCRL*, detected variants included missense (75.0%) and frame shift (25.0%), with 3 being previously unreported.

### Follow-up and kidney function

Two patients were lost to follow-up, leaving 21 patients for analysis. The median follow-up duration was 3.8 ± 3.15 years, and the median age of patients at the last follow-up was 9.8 ± 4.6 years. During this period, hypercalciuria was observed in 3 patients, nephrolithiasis occurred in 7 patients, and nephrocalcinosis was detected in 6 patients. AKI occurred in 8 patients, including 6 DD1cases and 2 DD2 cases, 3 of which were caused by infection. Progression to CKD was documented in 7 patients (Table 3): 6 with DD1 and 1 with DD2. CKD stages were distributed as follows: 4 patients at stage II, 2 at stage III, and 1 at stage V, and all these patients had an AKI history. Ultrasound results depicted a reduction in kidney size in 2 patients. Among 7 patients, 3 had minimal change disease, 1 had mesangial proliferative glomerulonephritis, and 1 had focal segmental glomerulosclerosis.

**Table 3:** Clinical data of patients presented with early renal failure during their childhood at the last follow-up

Patients ID	Dent type	Age of CKD onset	Age at the last follow-up	Serum creatinine (umol/L)	Urine protein/ creatinine ratio (ug/mg)	Urine albumin/ creatinine ratio (ug/mg)	Hypercalciuria	AKI	CKD	eGFR(mL/min/1.73 m²)	Renal ultrasound
4	DD1	7	9	140	-	-	Yes	Yes	CKD3	36.30	Nephrolithiasis, nephrocalcinosis, enhanced renal parenchymal echo
6	DD1	5	10	83	1365.18	344.1	Yes	Yes	CKD2	61.65	Nephrolithiasis, nephrocalcinosis, enhanced renal parenchymal echo
7	DD1	8	10	60	2228.89	410.5	Yes	Yes	CKD2	85.17	Nephrolithiasis, nephrocalcinosis, enhanced renal parenchymal echo
8	DD1	6	13	377	1845.77	1018.2	Yes	Yes	CKD5	13.80	Nephrolithiasis, nephrocalcinosis, small kidneys, enhanced renal parenchymal echo
9	DD1	7	7	65	1852.34	325.3	Yes	Yes	CKD2	68.78	Nephrolithiasis, nephrocalcinosis, enhanced renal parenchymal echo
13	DD1	5	7	65	-	323.5	Yes	Yes	CKD2	64.00	Nephrolithiasis, nephrocalcinosis,
22	DD2	15	20	334	1009.68	244.2	Yes	Yes	CKD3	58.90	Nephrolithiasis, small kidneys

DD1: dent disease type 1; DD2: dent disease type 2; AKI: acute kidney injury; CKD: chronic kidney disease

The clinical phenotypes in our cohort were compared with those reported in European populations at the last follow-up. Compared with European patients with DD1, the present study demonstrated a significantly higher incidence of nephrolithiasis (55.56% vs. 13.19%, *P* < 0.001) but a lower prevalence of nephrocalcinosis (33.33% vs. 60.69%, *P* = 0.039). No significant differences were observed in other clinical features (Table 4).

**Table 4:** Phenotype of patients with DD1 in the European populations and in our cohort at the last follow-up

Phenotype	European populations < 18 years [15] (n = 94)	This study < 18 years old (n = 18)^a^	*P*
Age at diagnosis (years; median, [IQR])	4 (1–7)	5.0 (3–9)	-
Nephrolithiasis, n (%)	12/91 (13.19%)	10/18 (55.56%)	<0.001
Nephrocalcinosis, n (%)	50/82 (60.98%)	6/18 (33.33%)	0.039
Age at last follow-up, years (median, IQR)	8.0 (5–12.0)	8.5 (5–10.5)	-
Serum creatinine, mg/dL (mean ± SD)	0.69 ± 0.38	0.79 ± 0.92	-
Hypercalciuria, n (%)	36/69 (52.17%)	13/18 (66.67%)	0.301
Urine albumin/ creatinine ratio , ug/mg (median, IQR)	270.0 (82.0–502.0)	323.5 (138.1–384.8)	-
CKD2-5, n (%)	44/88 (50.00%)	6/18 (33.33%)	0.300

DD1: dent disease type 1; CKD: chronic kidney disease; a: at the last follow-up; 18 patients were younger than 18 years of age and 1 patient was older than 18 years

### Clinical phenotypes and genotypes comparison in patients with DD1

Among patients with DD1, clinical phenotypes and genotypes were compared between those who developed chronic renal failure and those who did not. Significant differences were noted in nephrolithiasis (100% vs 30.76%, *P* = 0.011), nephrocalcinosis (85.33% vs 15.38%, *P* = 0.010), and AKI (100% vs 0%, *P* < 0.001) incidence between the two groups (Table 5). Moreover, proteinuria significantly increased in patients with CKD. No evidence of genotype-phenotype correlation was found between variant severity groups (severe vs moderate) and CKD progression.

**Table 5:** Comparison of the CKD patients with normal renal function among DD1 patients at the last follow-up

	CKD (n = 6)	Non-CKD (n = 13)	*P*
Age of CKD onset, years (median, IQR)	6.5 (5.0–7.25)	8.0 (4.5–12)	0.42
Nephrolithiasis, n (%)	6 (100%)	4 (30.76%)	0.011
Nephrocalcinosis, n (%)	5(83.33%)	2 (15.38%)	0.010
Hypercalciuria, n (%)	6 (100%)	6 (46.15%)	0.109
Urine protein/creatinine ratio, ug/mg (median, IQR)	1849.1 (1485.3–2134.8)	822.95 (497.7–1339.8)	0.028
Urine albumin/ creatinine ratio, ug/mg (median, IQR)	344.1 (324.4–714.4)	234.4 (93.65–357.4)	0.075
Severe variants, n (%)	4 (66.67%)	8 (61.54%)	1.000
nonsense	2 (33.33%)	3 (23.08%)	-
frameshift	2 (33.33%)	3 (23.08%)	-
large deletion	0 (0%)	1 (7.69%)	-
splicing mutation	0 (0%)	1 (7.69%)	-
Moderate variants, n (%)	2 (33.33%)	5 (38.46%)	1.000
missense	1 (16.67%)	5 (38.46%)	-
in-frame deletion	1 (16.67%)	0 (0%)	-
AKI, n (%)	6 (100%)	0 (0%)	<0.001

DD1: dent disease type 1; Severe variants: nonsense, frameshift, large deletion, or splice-site variants; Moderate variants: missense and in-frame deletion; AKI: cute kidney injury; CKD: chronic kidney disease

## Discussion

DD has been widely described across Asia, North America, and Europe. However, there is limited literature detailing the progression of CKD during childhood. This study is the first to describe the progression of DD from onset to CKD in pediatric patients while analyzing potential contributing factors.

First, we described the clinical phenotypes of 23 patients in this cohort. All enrolled cases manifested LMWP, consistent with previous studies [15, 16, 22]. However, not all patients exhibit hypercalciuria, nephrolithiasis, or nephrocalcinosis, therefore reflecting clinical manifestations heterogeneity. Additional symptoms, such as hypophosphatemia, rickets, glucosuria, hematuria, hypokalemia, nephrotic-range proteinuria, and kidney failure, were observed. Interestingly, nephrolithiasis prevalence in our cohort was significantly higher than that reported in European cohorts, highlighting potential regional or genetic differences. Previous studies suggest that podocyte damage may contribute to renal function decline in DD [5]. However, the renal biopsies limited availability in our cohort precluded detailed statistical analysis of podocyte involvement. Further studies with larger sample sizes and expanded histological data are required to explore this association.

To date, 397 and 378 distinct pathogenic variants in *CLCN5* and *OCRL* have been identified in HGMD, respectively, with truncating variants (nonsense and frameshift) accounting for 43% and 40% of these variants, followed by non-truncating and splicing variants. Our study identified 16 previously unreported pathogenic variants, expanding the DD genetic spectrum. Notably, missense, nonsense, and frameshift variants dominated the *CLCN5* variants in our cohort, whereas *OCRL* variants included both missense and frameshift variants. These findings underscore the advanced genetic testing and next-generation sequencing utility in diagnosing and classifying DD, facilitating personalized management strategies [4, 23]. The lack of genotype-phenotype correlation in DD poses challenges in predicting disease severity. However, specific *CLCN5* variants including those reported in rapidly progressive cases [24], may require further investigation to identify genetic predictors of an aggressive disease course. Early genetic screening in symptomatic families and anticipatory guidance for affected individuals may improve outcomes.

DD is a hereditary condition often associated with nephrolithiasis and CKD [25]. While CKD in DD typically progresses slowly, male patients with DD1 commonly develop ESRD between the ages of 30 and 50 years [26, 27]. However, some patients may never progress to ESRD during their lifetime. Consequently, many individuals remain undiagnosed until they present with symptomatic kidney failure, often in the third to fourth decades of life. An observational cohort study on genetic tubulopathies impacting kidney function identified 116 patients with kidney failure out of 885 with available data. Among these, the majority were diagnosed with DD (31%) and distal renal tubular acidosis (20%), with renal prognosis appearing particularly poor in familial hypomagnesemia with hypercalciuria and nephrocalcinosis, as well as in DD [28]. The pathogenic mechanisms driving CKD in DD remain unclear. Conversely, our cohort demonstrated CKD onset during childhood, highlighting an accelerated disease course. Progression to CKD in our cohort was strongly associated wit h nephrolithiasis, aligning with previous findings [23]. Patients with abnormal renal function had a significantly higher incidence of stone events than those with normal renal function. Therefore, early identification and management of nephrolithiasis may be critical in delaying CKD progression.

Furthermore, our study found a higher prevalence of AKI in patients with abnormal renal function. AKI contributes to structural and functional kidney damage, thereby increasing CKD development risk [29]. Both AKI episode severity and frequency are key factors affecting the transition from AKI to CKD [30]. Accordingly, preventive strategies targeting AKI could play a crucial role in mitigating CKD progression in patients with DD. Notably, a statistically significant association was demonstrated between CKD progression and nephrocalcinosis in patients with DD1, suggesting a possible association with CKD progression. Previous studies have indicated that nephrocalcinosis may contribute to CKD progression [31]. These findings highlight the need for further research to better understand nephrocalcinosis role in CKD progression and to develop targeted interventions.

This study has several limitations. Notably, the relatively small cohort size, particularly for subgroup comparisons among patients with DD1, reduced the statistical power and generalizability of the results. Additionally, the short follow-up period constrained the ability to observe long-term outcomes. Despite these limitations, our findings revealed significant phenotypic differences from previous cohorts, particularly the early onset of CKD in childhood. Heterogeneity in clinical presentations also highlights the potential for misdiagnosis as nephrotic syndrome, leading to inappropriate immunosuppressive treatments. Currently, treatment options for DD are limited. Although thiazide diuretics can reduce hypercalciuria and nephrolithiasis, their role in slowing CKD progression is modest. Developing targeted therapies addressing the underlying pathophysiology of *CLCN5* and *OCRL* variants could revolutionize care for DD.

## Conclusions

In summary, our study expands the genetic spectrum of DD and highlights the potential for CKD progression during childhood. Nephrolithiasis, nephrocalcinosis, and AKI are indicators of a more severe CKD phenotype, emphasizing the need for early diagnosis and proactive management. Further research on genotype-phenotype correlations and targeted therapies development is essential to improve outcomes for patients with DD.

## Data Availability

The datasets generated or analyzed in this study are also available from the corresponding author upon reasonable request.

## Abbreviations

ESRD: End-stage renal disease
CKD: Chronic kidney disease
LMWP: Low-molecular-weight proteinuria
DD: Dent disease
DD1: Dent disease type 1
DD2: Dent disease type 2
KDIGO: Kidney Disease Improving Global Outcomes
AKI: Acute kidney injury
